# Characteristics and prediction of agricultural ecological efficiency for coordination between economy and environment

**DOI:** 10.1371/journal.pone.0302971

**Published:** 2024-05-30

**Authors:** Lijun Ma, Fengyu Guo, Zhaoya Chen, Jingyi Meng, Lei Xu, Shi Yin

**Affiliations:** 1 College of Land and Resources, Hebei Agricultural University, Baoding, China; 2 College of Urban and Rural Construction, Hebei Agricultural University, Baoding, China; 3 College of Geography and Tourism, Baoding University, Baoding, China; 4 College of Economics and Management, Hebei Agricultural University, Baoding, China; East China Normal University, CHINA

## Abstract

Agricultural ecological efficiency is an important tool with which to measure the coordination of the sustainable development of agricultural economies and ecological environments. In this paper, a super-efficiency slacks-based measures model was used to measure the agricultural ecological efficiency in Hebei Province. The characteristics of spatial and temporal evolution patterns were explored using a spatial Markov transfer matrix. The results showed that (i) based on measurements, the agricultural ecological efficiency in Hebei Province showed regional differences in four regions (eastern, northern, central and southern Hebei) and 141 counties; (ii) from the perspective of evolutionary characteristics of agricultural ecological efficiency, the overall development of in Hebei Province was good, with more concentrated spatial distribution and more obvious direction, while the type of transfer of agricultural ecological efficiency in Hebei Province showed strong stability that was significantly affected by geographical neighborhood conditions and the club convergence phenomenon; (iii) from the perspective of the long-term evolutionary trend of agricultural ecological efficiency, the areas adjacent to counties with low efficiency had limited potential for improvement, and the areas adjacent to counties with high grade had great potential. However, it was difficult to achieve large-scale improvement in agricultural ecological efficiency in Hebei Province, whether the impact of geospatial backgrounds was considered or not.

## 1. Introduction

Agriculture, rural areas and farmers are an important foundation for national stability and security. Agriculture plays a decisive role in developing the national economy and ensuring food security, and is the foundation for the survival of a country and a nation. Land is the most basic resource for human survival and development. For agricultural development and rural construction, farmers’ lives are an essential link, and the sustainable use of land resources and sustainable agricultural development complement and influence each other. Handling the relationship between agricultural development and land management is the key to solving the problem of three rural areas and farmers. Since the founding of the People’s Republic of China, China has carried out many rural land system reforms in order to adapt to the development level of rural productivity. These reforms have achieved very remarkable results. For example, the household contract responsibility system greatly stimulated the enthusiasm of farmers, improved agricultural productivity and improved the living standards. The proposal and implementation of three rights separation will revitalize rural land, expand agricultural investment and meet the needs of agricultural modernization. Today’s economic development and urbanization have led to the reduction of the quantity and quality of cultivated land, while the process of industrialization has normalized extensive agricultural production in combination with high investment, high energy consumption and high pollution. Social development, measured by economic levels only, does not meet the requirements of the current development era. The model of agricultural development, at the cost of excessive consumption of natural resources and destruction of the ecological environment, is not sustainable. It is imperative to change the agricultural production mode and realize agricultural transformation and development. To this end, the 18th National Congress of the CPC included ecological civilization construction; in 2015, important measures of green agriculture development were focused on organic and ecological agriculture. The 19th National Congress positioned ecological civilization construction as the sustainable development of the Chinese nation, formally proposed the rural revitalization strategy and raised agricultural and rural development to an unprecedented height. Therefore, it is of great significance to explore new paths to sustainable agricultural development for the rational utilization and scientific management of land resources.

Ecological efficiency has been recognized by foreign scholars [1]; it has been proposed and defined, as given by Claude Fussler [2]. Thus, China began to study ecological efficiency. Agricultural ecological efficiency is the introduction of ecological elements to improve agricultural production efficiency, quantitatively representing the coordination of social economies and ecological environments to facilitate regional development [3]. It focuses on realizing maximum social, economic and ecological values while reducing environmental input and negative environmental output. The core of ecological efficiency must take into account the dual dimensions of economy and ecology in the evaluation process [4]. This is more in line with current social requirements for the sustainable utilization of resources, sustainable development and ecological civilization construction. Domestic scholars have researched agricultural ecological efficiency only recently, but have achieved fruitful results. Regarding research methods, the evaluation of ecological efficiency can be accomplished using the ratio method [5], life cycle method [6], ecological footprint method [7], stochastic frontier analysis method [8,9] and data envelope analysis method, among others [10]. However, the data envelope analysis method is the most commonly used. As research efforts have increased, these methods have been expanded and improved based on data envelope analysis; for instance, Guo used the ultra-efficiency DEA model to measure the industrial ecological efficiency of six central provinces over 11 years and found that industrial ecological efficiency first and decreased and then rose, with low overall levels and small regional differences [11]. Wu et al. used a three-stage DEA model, introducing environmental variables and eliminating the influence of environmental factors and random error on the ecological efficiency of agricultural land in Henan Province. They proved the influence mechanism of environment and location factors on the ecological efficiency of Henan Province [12]. Pan et al. used a slacks-based measures (SBM) model to measure the efficiency of agricultural ecology in China from 1998 to 2009 and proposed ways to improve efficiency, e.g., reducing resource consumption and pollutant emission [13]. Most research has focused on three aspects of agricultural ecological efficiency: evaluation index construction, spatial and temporal evolution characteristics and analysis of influencing factors. At present, the evaluation of agricultural ecological efficiency mainly utilizes evaluation index systems, i.e., input and output [14]. This research on agricultural ecological efficiency in China began late, but in the process, the negative environmental impacts of agricultural production processes became clear, and these negative impacts were included in the index system as an undesired output. Agricultural carbon emission and agricultural pollution were selected as undesired outputs to be incorporated into the agricultural ecological efficiency evaluation system [15]. Agricultural production processes not only emit carbon dioxide, but also absorb it, so some scholars include carbon sink into desired outputs, with carbon emissions sorted into undesired outputs. The input index was selected from the aspects of agricultural capital, labor force, natural resources, chemical products, mechanization and so on. It took economic benefit and carbon sink efficiency as expected outputs, and carbon emission as the unexpected index, to establish the agricultural ecological efficiency evaluation index system of the Yangtze River Economic Belt [16]. As one of the current research hotspots, the analyses of many scholars in early research studies focused on research unit spatial and temporal differences [17,18]. Yang used structure to measure the efficiency from the perspective of time changes and spatial patterns in Chongqing [19]. The results showed that the overall land use structure efficiency level was medium, but the situation grew more optimistic and was rising annually. Furthermore, spatial differences were significant. However, most of the research was relatively macroscopic; time, space and dimension were not close.

The first law of geography put forward the idea that the spatial distribution of geographical objects or the properties of that spatial distribution may have mutual relationships. Thus, regional spatial correlation and spatial and temporal dynamic evolution aroused the attention of scholars. Scholars began to pay attention to spatial correlation and dynamic evolution between regions [20,21]. According to existing research results [22], China’s ecological efficiency research mostly takes the form of administrative areas known as units. These research units are connected to each other and have relatively obvious spatial relationships. Ren took the years 2006, 2010 and 2018 as Study Period 14, in which the spatial correlation of ecological efficiency in 153 counties in the Beijing-Tianjin-Hebei region was tested [23]. The research results showed that there was significant and positive spatial correlation and continuous change over time. Therefore, it was deemed very necessary to conduct a study of the spatial and temporal distribution and evolution characteristics. Foreign research on the influencing factors of agricultural efficiency began early, and different scholars chose different influencing factors based on different research perspectives. For instance, Kawagoe considered the influence level of labor productivity in areas with similar economic development level, determining that it was limited [24]. Helfand studied the factors influencing agricultural technical efficiency in central Brazil and showed that modern inputs and farm sizes were determinants and important factors, respectively [25]. Wadud investigated the production efficiency of farmers in Bangladesh and showed the influence of scale efficiency [26].

Domestic scholars on factors affecting agricultural ecological efficiency are still in the initial stage. Most use geographical detectors [27], logistic regression analysis [28] and random-effects panel tobit models [15] or explore other methods. In a study of agricultural ecological efficiency in 43 rural fixed observation points in Fujian province, 20 indicators were selected including location conditions, economic development level, agricultural development, agricultural management subject, human resources structure, system, and technical level. Then, using a cross-section timing model for comparative analysis, the researchers explored the leading factors to provide reference for efficiency improvement efforts [29].

Throughout the relevant research, both at home and abroad, strides have been made in comprehensive evaluation of the spatial and temporal evolution characteristics and influencing factors of agricultural ecological efficiency. The research was carried out layer by layer on the national, provincial and municipal levels, which essentially defined the theoretical connotation of agricultural ecological efficiency. Evaluation methods were more scientific and reasonable, and the evaluation indicators were gradually enriched, laying a solid foundation for subsequent in-depth studies of agricultural ecological efficiency. Still, some shortcomings remain in the existing research; for instance, Hebei Province is a major agricultural province, an important agricultural production and processing base in North China. However, there have been relatively few studies on agricultural ecological efficiency in Hebei Province. Additionally, the construction of the evaluation index system for agricultural ecological efficiency needs further improvement, especially on the output end. Compared with previous studies, the existing literature has focused more on undesired outputs, such as agricultural carbon emissions, while ignoring the expected ecological value output in the agricultural production process. Integrating agricultural ecological value factors into the evaluation index system to break the shackles of past measures of grain output and agricultural output would be of value, but such studies are still relatively rare. Additionally, there have been few current studies on spatial and temporal evolution characteristics of agricultural ecological efficiency; more time series evolution and spatial pattern evolution analysis from a macroscopic perspective are called for. There is a lack of study of the combination of spatial and temporal evolution characteristics of macroscopic agricultural ecological efficiency and microscopic spatial and temporal evolution law. Lastly, it is rare to discuss future evolution trends in agricultural ecological efficiency.

Future work of value would include: carrying out research on agricultural ecological efficiency in Hebei Province, exploring ways to maximize agricultural output with minimum resource consumption and pollution, while ensuring the quality of agricultural products [30]. It is of far-reaching significance to realize the sustainable development of agriculture in the future. It could even provide a reference for exploring how to coordinate the relationship between social and economic development, ecological and environmental protection and resource conservation and intensive utilization in Hebei Province. Therefore, this paper was based on panel data of 141 counties in Hebei Province, with agricultural carbon emissions as undesired output and farmland ecosystem service value as expected ecological output. The goal was to measure agricultural ecological efficiency in Hebei Province, analyze spatial and temporal distribution patterns, construct a traditional Markov transfer probability matrix, expand it to the space dimension (with respect to agricultural ecological efficiency in Hebei Province) and predict long-term evolution trends in order to make up for the shortcomings of current research.

**Fig 1:**
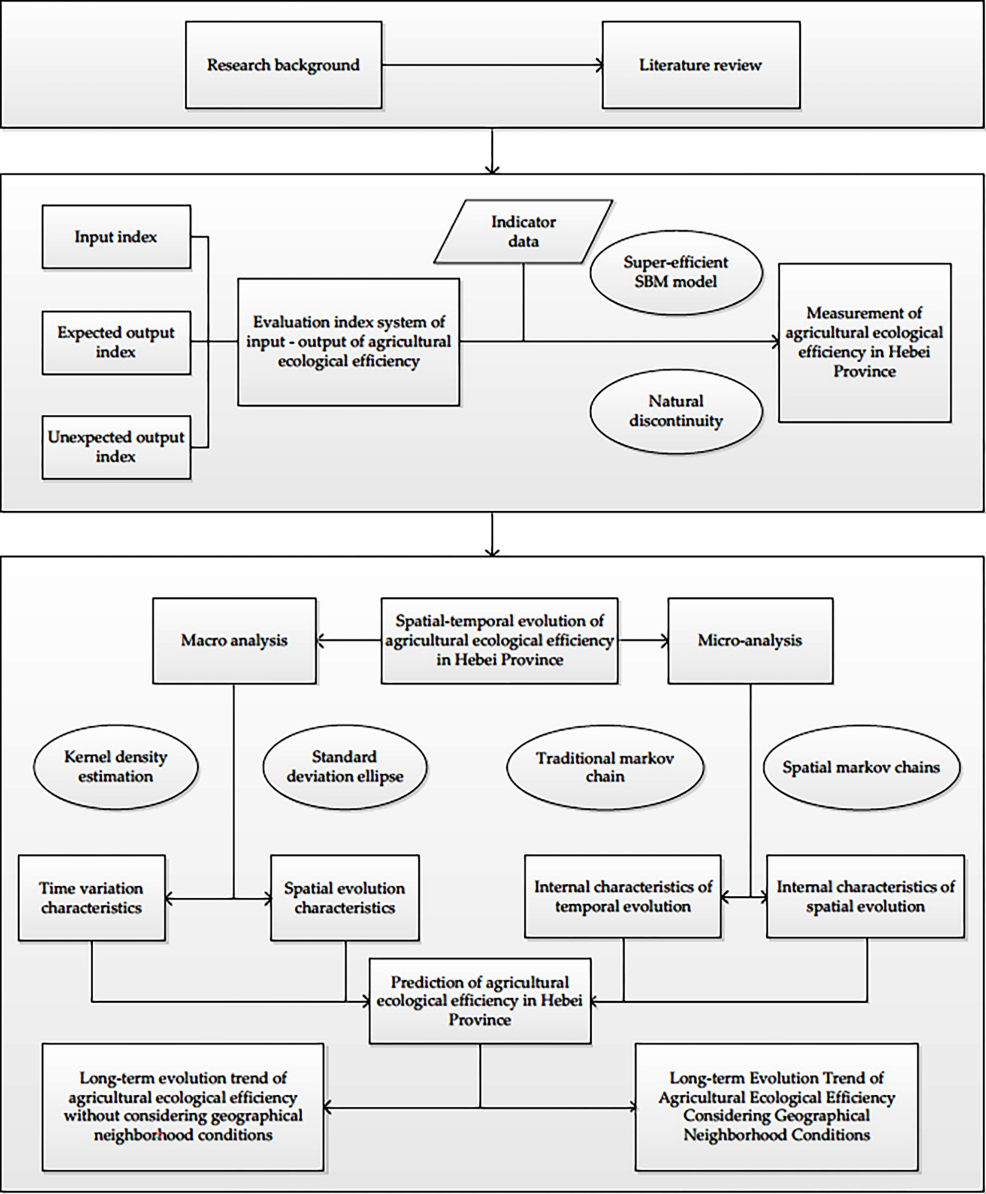
Research ideas.

As shown in Fig 1, the research was based on a background and literature review, using input, expected output, and three angles of expected output to build an index system for the evaluation of agricultural ecological efficiency. A superefficient SBM model and natural discontinuities were used to determine whether agricultural ecological efficiency measures worked in Hebei Province. On the basis of measurement results, the temporal and spatial evolution of agricultural eco-efficiency in Hebei Province was analyzed on macroscopic and microscopic scales. Kernel density estimation and standard deviation ellipses were used to analyze the temporal and spatial evolutionary characteristics of agricultural eco-efficiency in Hebei Province. Additionally, microscopic analysis, based on the traditional Markov chain, was used to separately analyze Hebei’s agricultural ecological efficiency and temporal and spatial evolution characteristics. Finally, based on the results of these analyses, considering geographical areas but not considering geographical field conditions, respectively, long-term evolutionary trend predictions were created for Hebei Province.

## 2. Study area and index construction

### 2.1. Overview of the study area

Hebei Province is located in the North China Plain, near Beijing and Tianjin, the Bohai Sea in the east, Taihang Mountain in the west, and Shandong, Henan, Shanxi, Inner Mongolia and Liaoning. It has a total area of 188,800 km^2^. It has landform types—including plateau, mountain, basin, hilly, plain, lake and other landforms—suitable climate, distinct seasons, and many rivers. By the end of 2019, the permanent resident population of Hebei Province was 75.92 million. The non-agricultural population accounted for 57.62% of the total population (urbanization rate), and the annual sown area of grain was 6,389 thousand hm^2^. The total grain output was 37. 959 million t.

### 2.2. Data source

The vector administrative boundary map was obtained from the National 1:1 million basic geographic database in 2017, published by the National Basic Geographic Information Center (www. webmap. In cn), GS (2016) 2556. Considering spatial connectivity and data accessibility, 141 counties in Hebei Province were selected as study areas.

This study collected panel data on 9 indicators in 141 counties in Hebei Province. Social and economic data and agricultural production data were from 2006–2019, including Shijiazhuang, Tangshan, Qinhuangdao, Handan, Xingtai, Baoding, Zhangjiakou, Chengde, Cangzhou, Langfang and Hengshui prefectures.

### 2.3. Indicator construction

The input-output evaluation index system for agricultural ecological efficiency in Hebei Province was constructed using or referring to the following: ideas and methods of selecting ecological efficiency indicators used by domestic and foreign scholars [23,31,32]; understanding of agricultural eco-efficiency and other theories; the principles of quantification; the availability and rationality of data; existing agricultural eco-efficiency input-output index systems [15,22,33]; and the actual situation in Hebei Province, taking economy [34] and ecology [7] as the foothold (Table 1). Input indicators were selected as follows: aspects of land, labor, energy and resources, crop-sown area, number of agricultural employees, total power of agricultural machinery, rural electricity consumption, and effective irrigation area represented land input, labor input, energy input and water resources input, respectively. The expected output indicators were selected from social, economic and ecological aspects. Select agricultural output value, total grain output and farmland ecosystem service value were selected to measure economic output, social output and ecological output, respectively. Considering that the agricultural production process will have a certain negative impact on the environment, we selected agricultural carbon emissions to represent the undesired output. Due to software limitations, when measuring, this index did input processing.

**Table 1:** The input-output index system to evaluate agricultural ecological efficiency in Hebei Province.

Indicator type	Sub-index	name of index
**Investment index**	Land investment	Crop sown area (khm2)
	Labor input	Number of agricultural employees (people)
	Energy input	Total power force of agricultural machinery (104kW)
		Rural electricity consumption (104kW)
	Water input	Effective irrigation area (khm2)
**Expect output**	Economic output	Agricultural output value (10^4^Yuan)
	Social output	Total grain output (107kg)
	Ecological output	Farmland Ecosystem Service Value (10^4^Yuan)
**Undesired output**	carbon emission	Agricultural carbon emissions (107kg)

To correct the unit standard equivalent value in Hebei Province and obtain the farmland ecosystem service value of each unit and each year by calculation, we referred to existing literature [35,36]. Agricultural carbon emissions were mainly based on the use of chemical fertilizers, pesticides, agricultural film, diesel oil, land tillage and irrigation in the process of agricultural production, referring to Li [37]. The carbon emission coefficients used to calculate available carbon emissions were 0.8956 kg/kg, 4.9341 kg/kg, 5.18 kg/kg, 0.5927 kg/kg, 312.6 kg/km^2^ and 20.476 kg/km^2^.

## 3. Methodology

### 3.1. Ultra-efficiency SBM model

Data envelope analysis, referred to as DEA, is widely used because it has no weight setting, and because it reduces the impact of subjective factors on measurement results. However, the traditional DEA model has some deficiencies; it is not suitable for the unexpected output index, it ignores the input-output relaxation, and it cannot rank effective units. The super-efficient SBM model, proposed by Tone Kaoru and improved by Sahoo, solved the above deficiencies [38,39]. The model construction was as follows:

$$\begin{array}{l} \min\rho_{SE}=\frac{1+\frac{1}{m}\sum_{i=1}^{m}s_{i}^{-}/x_{ik}}{1-\frac{1}{s}\sum_{r=1}^{s}s_{r}^{+}/y_{rk}}\\ s.t.\{\begin{array}{l} \sum_{j=1,j\neq k}^{n}x_{ij}\lambda_{j}-s_{i}^{-}=x_{ik}\\ \sum_{j=1,j\neq k}^{n}y_{rj}\lambda_{j}+s_{r}^{+}=y_{rk}\\ \sum_{j=1,j\neq k}^{n}\lambda_{j}=1\\ \lambda ,s^{-},s^{+}\geq 0\\ i=1,2,\cdots ,m;r=1,2,\cdots ,q\\ j=1,2,\cdots ,n(j\neq k) \end{array} \end{array}$$ (1)

In the formula, a set of n decision units is assumed, denoted as DMUj. The value range of j is 1 to n, with m input elements and q output elements set for each DMUi, yr, i from 1 to m and j from 1 to q. x is the element in the matrix constructed by input elements; y is the element in the matrix constructed by output elements; X and Y are input and output moment matrixes; S—and S + represent relaxation variables of input-output, and λ is a column vector. ρSE is the value of agricultural ecological efficiency.

### 3.2. Kernel density estimation

Kernel density estimation is a nonparametric test method that originated from the discipline of probability theory. It is often used by various disciplines and fields to estimate unknown density functions. Kernel density estimation was employed herein, using the peak function to fit the existing data points and complete the real simulation of the probability distribution curve. Assuming that the density function of the random variable is f (x), for the random variable Y, it has n independent and identical observations, respectively. Assuming it has y1,y2,…, yn, then the estimator of the Kernel density function is:

$$f(x)=\frac{1}{nh}\sum_{i=1}^{n}K(\frac{y_{i}-y}{h})$$ (2)

$$K(x)=\frac{1}{\sqrt{2\Pi }}\exp(-\frac{x^{2}}{2})$$ (3)

where n is the number of sample observed values, h is the window width, or smooth parameter, K () is a weighted or smoothing function, and y is the mean. The size of the window width affects the smoothness of the estimated density function and, according to previous correlation studies, it takes h = cN-1/5(c is a constant).

### 3.3. Standard deviation ellipse

Standard deviation ellipse (SDE) is an analysis method that can accurately represent the spatial direction distribution pattern and characteristics of geographical elements [40]. It is used to characterize the overall spatial distribution of geographical elements through basic elements such as the oval center of gravity, long axis and short axis. Among these, the center of gravity can represent the relative position of the spatial distribution, the long half axis indicates the direction of the distribution in the study area and the short half axis indicates the dispersion of the data distribution—the smaller the ratio of the short and long half axes, the more obvious the directionality of the data. The calculation formula is as follows:

$$\begin{array}{c} {\bar{X}}_{w}=\frac{\sum_{i=1}^{n}w_{i}x_{i}}{\sum_{i=1}^{n}w_{i}};{\bar{Y}}_{w}=\frac{\sum_{i=1}^{n}w_{i}y_{i}}{\sum_{i=1}^{n}w_{i}}\\ \sigma_{x}=\frac{\sqrt{\sum_{i=1}^{n}{\left(w_{i}{\tilde{x}}_{i}\cos\theta -w_{i}{\tilde{y}}_{i}\sin\theta \right)}^{2}}}{\sum_{i=1}^{n}w_{i}^{2}}\\ \sigma_{y}=\frac{\sqrt{\sum_{i=1}^{n}{\left(w_{i}{\tilde{x}}_{i}\sin\theta -w_{i}{\tilde{y}}_{i}\cos\theta \right)}^{2}}}{\sum_{i=1}^{n}w_{i}^{2}} \end{array}$$ (4)

${\bar{X}}_{w}{\bar{Y}}_{w}$ In, (,) is the center of gravity coordinate of the study area; (xi、xj) is the spatial location element; wij represents the attribute weight; (yi、yj) represents the azimuth angle; and σx and σy represent the standard deviation along the x and y axes, respectively [41].

### 3.4. Markov chain

1. Traditional Markov chains and spatial Markov chains
  Markov chain is a stochastic process {*x(a)*,*a∈A*} with no aftereffect. If agroecological efficiency is divided into *N*(*N =* 4) states, it can form the state transition probability matrix of N rows and N columns, as shown in Table 2. Among, *P*_*ij*_ *= N*_*ij*_*/N*_*i*_, for instance, *P*_12_ *= N*_12_*/N*_1_ represents the probability of shifting from units with agroecological efficiency of state 1 to units with agroecological efficiency of state 2 at year t + 1.
2. Steady-state distribution of the Markov chains
  After a one-step or multistep state transfer, the system reaches an equilibrium state where the state probability distribution remains constant regardless of how many subsequent state transitions. This equilibrium state, which no longer changes with time and does not depend on the initial state, is called a steady-state distribution [42]. It also exists in the Markov chain, which can predict the future evolution of the Markov process.

**Table 2:** Markov chain state transfer probability matrix (N = 4).

t/t+1	1	2	3	4
**1**	P_11_	P_12_	P_13_	P_14_
**2**	P_21_	P_22_	P_23_	P_24_
**3**	P_31_	P_32_	P_33_	P_34_
**4**	P_41_	P_42_	P_43_	P_44_

According to previous studies [22,23], the transformation of agricultural ecological efficiency migration is not completely isolated. Geographical proximity to the spatial overflow of the region of the transition has certain influence. Therefore, compared with the traditional Markov probability transfer matrix, a spatial Markov chain was used to make up for the former’s influence on spatial background. This was more conducive to revealing the agricultural ecological efficiency and the internal connection between spatial and temporal evolution and geographical location. The traditional N row and N column state transition probability matrix is decomposed into an N row and N column state transition conditional probability matrix, according to the spatial lag type of the starting year (Table 3). The spatial lag type is divided according to the spatial lag value. The spatial lag value is the sum of the product of the constructed spatial weight matrix and the efficiency value of the county, and the formula is as follows:

$$Lag_{a}=\overset{n}{\underset{b=1}{\sum }}Y_{b}W_{ab}$$ (5)

where *Laga* is the spatial lag value of the county a, *Yb* is the observed value of the county *b*, *n* is the total number of counties and *Wab* is the spatial weight matrix, representing the spatial relationship of county a and county b.

**Table 3:** Spatial Markov chain state transfer conditional probability matrix (N = 4).

Space lag	t/t+1	1	2	3	4
**1**	1	P_11/1_	P_12/1_	P_13/1_	P_14/1_
	2	P_21/1_	P_22/1_	P_23/1_	P_24/1_
	3	P_31/1_	P_32/1_	P_33/1_	P_34/1_
	4	P_41/1_	P_42/1_	P_43/1_	P_44/1_
**2**	1	P_11/2_	P_12/2_	P_13/2_	P_14/2_
	2	P_21/2_	P_22/2_	P_23/2_	P_24/2_
	3	P_31/2_	P_32/2_	P_33/2_	P_34/2_
	4	P_41/2_	P_42/2_	P_43/2_	P_44/2_
**3**	1	P_11/3_	P_12/3_	P_13/3_	P_14/3_
	2	P_21/3_	P_22/3_	P_23/3_	P_24/3_
	3	P_31/3_	P_32/3_	P_33/3_	P_34/3_
	4	P_41/3_	P_42/3_	P_43/3_	P_44/3_
**4**	1	P_11/4_	P_12/4_	P_13/4_	P_14/4_
	2	P_21/4_	P_22/4_	P_23/4_	P_24/4_
	3	P_31/4_	P_32/4_	P_33/4_	P_34/4_
	4	P_41/4_	P_42/4_	P_43/4_	P_44/4_

Supposing that the Markov probability transition matrix is the _ {_πi,i = 1,2,…}, pij, for a one-step transfer probability matrix, if *πp* = *π*, then:

$$\begin{array}{c} \sum_{i=1}^{N}\pi_{i}p_{ij}=\pi_{j};\;j=1,2,\ldots ,N\\ \sum_{i=1}^{n}\pi_{i}=1\\ 0\leq \pi_{i}\leq 1 \end{array}$$ (6)

where *πi* denotes the steady-state distribution of the Markov chain. Similarly, this steady-state distribution can also be applied to the spatial Markov chain; that is, the stationary state is calculated by the above principle under different spatial lag states.

## 4. Results and discussion

### 4.1. Results

**Fig 2:**
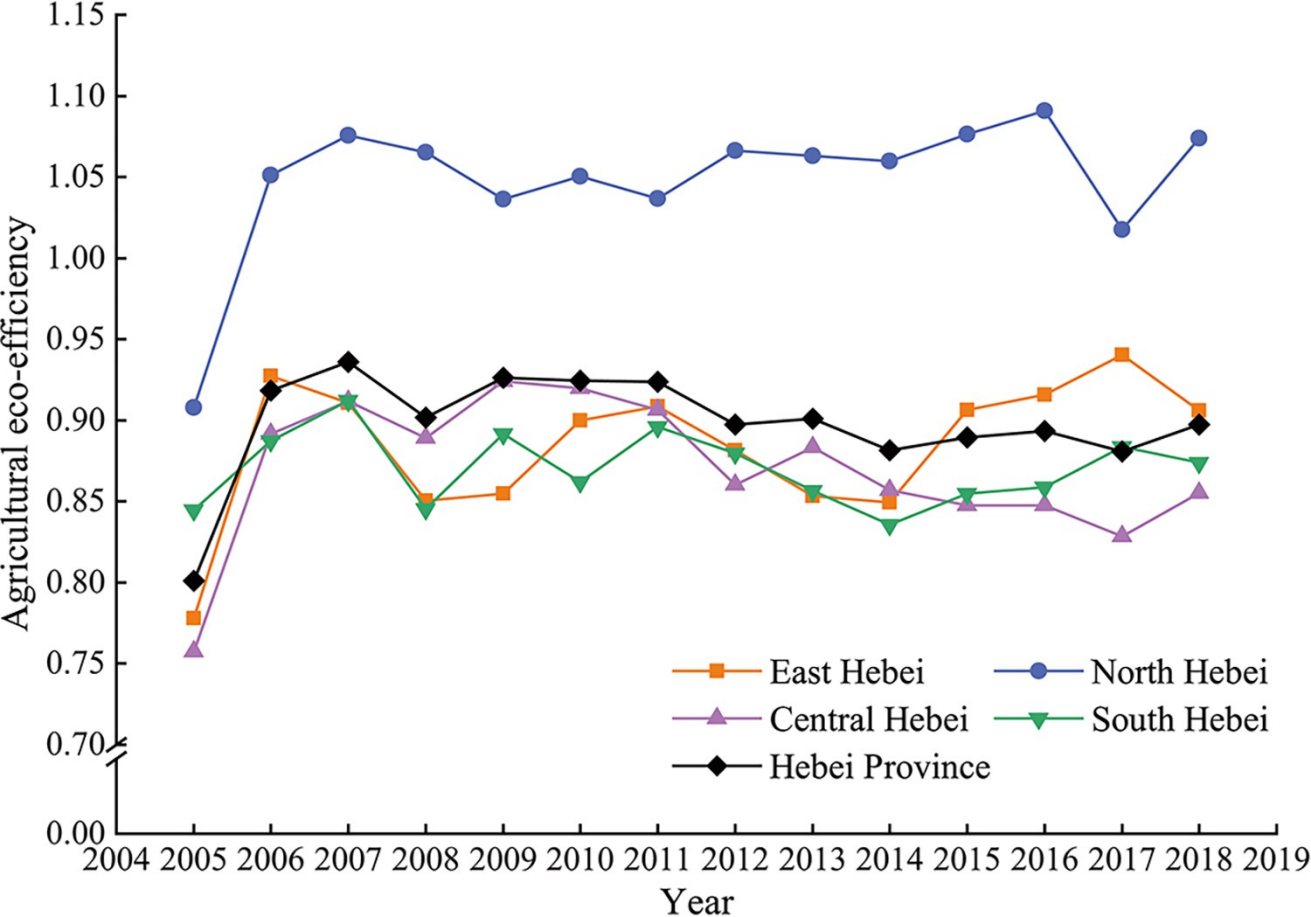
Agricultural ecological efficiency in Hebei Province.

This paper used the DEA Solver Pro5. 0 software and an ultra-efficiency SBM model to measure the agricultural ecological efficiency of Hebei Province. As shown in Fig 2, as far as the whole Hebei Province is concerned, the agricultural ecological efficiency first rose and then slightly declined, with an overall rising trend. Trends showed increasing agricultural ecological efficiency in Hebei Province compared with the initial stage of research.

In 2004, the central file focused on the three rural, agricultural taxes and fees reform, favorable policies to mobilize the enthusiasm of farmers, increase agricultural investment and improve agricultural production capacity. These favorable conditions led to 2005, during which agricultural ecological efficiency averages improved and reached the levels of the 2007 research period. At the same time, it also motivated farmers to increase their use of land fertilizer and pesticides, as well as agricultural plastic film. Although, to a certain extent, these improved agricultural output, they also led to soil pollution, water pollution and ecological environment pollution. These, unchecked, would inevitably cause the decline of agricultural ecological efficiency. During 2007–2014, in Hebei Province, agricultural ecological efficiency showed small fluctuations in the cause of the decline. However, the pursuit of solely economic benefits from agricultural development is doomed to be unsustainable. With the development of social economy and the changing in ideas, under the strategic background of two-type agriculture and ecological civilization construction, more attention must be paid to the efficient use of resources. This must be done at an individual, societal, and national level, in order to ensure ecological, environmental, and agricultural (food) security. Thus, in the 2014–2018 period, Hebei Province showed a rising trend in agricultural ecological efficiency.

Hebei Province can be divided into counties in eastern Hebei (Tangshan and Qinhuangdao), northern Hebei (Zhangjiakou, Chengde), central Hebei (Shijiazhuang, Baoding, Cangzhou, Hengshui and Langfang), and southern Hebei (counties under Handan and Xingtai). As can be seen from Fig 2, the average agricultural ecological efficiency in northern Hebei has been in an effective state since 2006, and is significantly higher than that observed in eastern, central and southern Hebei. In the initial stage of the study, the average agricultural ecological efficiency in eastern, northern, central and southern Hebei was 0.7779, 0.9078, 0.7573 and 0.84454, respectively. The order was as follows: northern Hebei region> southern Hebei region> eastern Hebei region> central Hebei region. At the end of the study, the average agricultural ecological efficiency values in eastern, northern, central and southern Hebei were 0.9060, 1.0739, 0.8551 and 0.8738, respectively, ranked as follows: northern Hebei> eastern Hebei> central Hebei> southern Hebei. As shown in Table 4, comparing the end of the study to early data, Hebei agricultural ecological efficiency grew fast, reaching 18.30% in the Jidong region and 16.46% for the eastern Hebei. The agricultural ecological efficiency growth rate for the central Hebei region was 12.91%, while the slowest growth rate was observed in the southern Hebei region, with only 3.47%.

**Table 4:** Growth rate of agricultural ecological efficiency in Hebei Province.

	2005	2018	Speed of increase
**Eastern Hebei area**	0.77792	0.906	16.46%
**Northern Hebei region**	0.90782	1.07394	18.3%
**Central Hebei region**	0.75734	0.85511	12.91%
**Southern Hebei region**	0.84454	0.87383	3.47%

**Fig 3:**
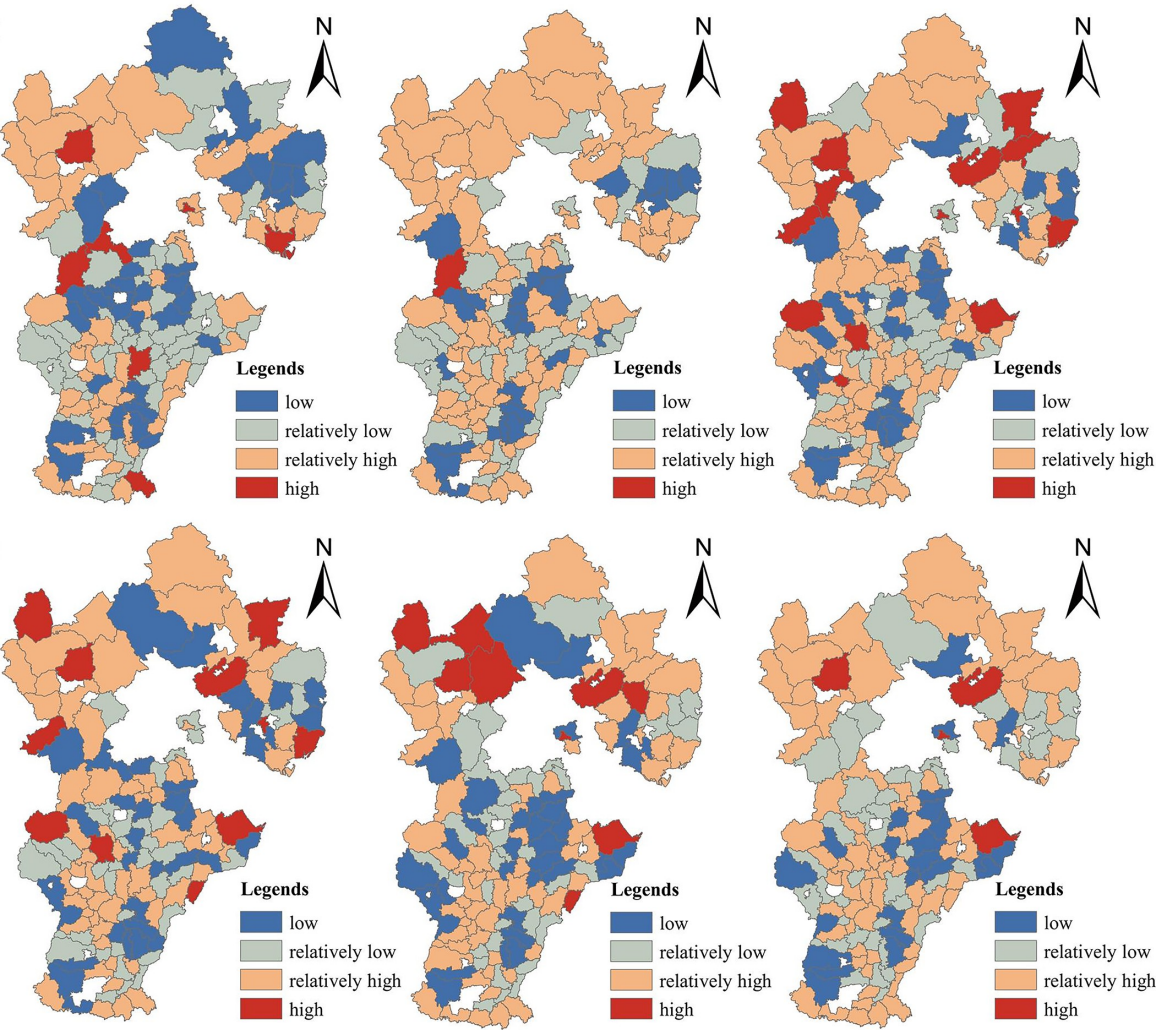
Spatial distribution diagram of agricultural ecological efficiency in Hebei Province.

**Fig 4:**
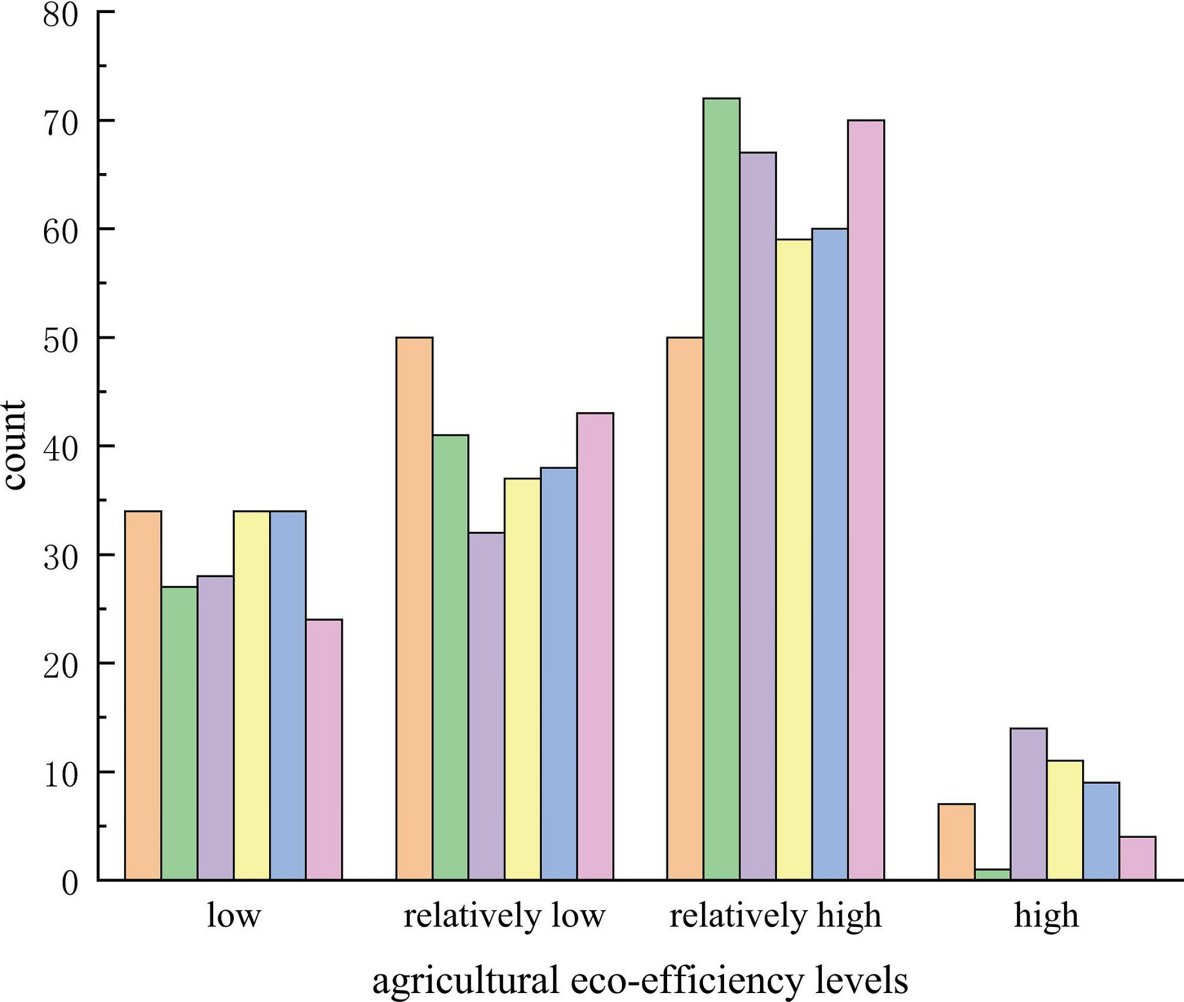
Distribution diagram of agricultural ecological efficiency grades in Hebei Province.

In order to intuitively understand the spatial distribution of agricultural ecological efficiency in Hebei Province, this paper selected 2005, 2008, 2011, 2014, 2017 and 2018 as time nodes, using ArcGIS 10.3 software. According to the natural breakpoint method, agricultural ecological efficiency values were sorted into four grades—relatively low, low, relatively high, high—generating agricultural ecological efficiency data for Hebei Province, as well as spatial distribution and change, as shown in Figs 3 and 4.

As shown in Fig 3, a relatively small number of counties were rated highly efficient in the study period. Efficiency was consistently sporadic in northern Hebei and eastern Hebei, where we observed the highest number of relatively high- and high-rated counties. This was slightly higher than the number of lower-rated counties; half or more of all counties in Hebei Province, in fact, mostly distributed in eastern and northern Hebei. Through the above analysis, it can be shown that counties, especially under the same prefecture-level city, had closer regional communication, mutual drive and mutual influence. However, counties in Hebei Province still have significant room for improvement, with respect to agricultural ecological efficiency.

According to Fig 4, the number of low-grade counties among the 141 counties in Hebei Province decreased by 10 over the study period. The agricultural ecological efficiency in Hebei Province showed the development of hierarchical aggregation, with the number of low grade, medium-low grade and high grade counties decreasing, while the number of middle and high grade counties increased significantly.

### 4.2. Discussion of spatiotemporal evolution characteristics

#### 4.2.1 Analysis of macroscopic spatial-temporal evolution

(i) Time-series analysis

**Fig 5:**
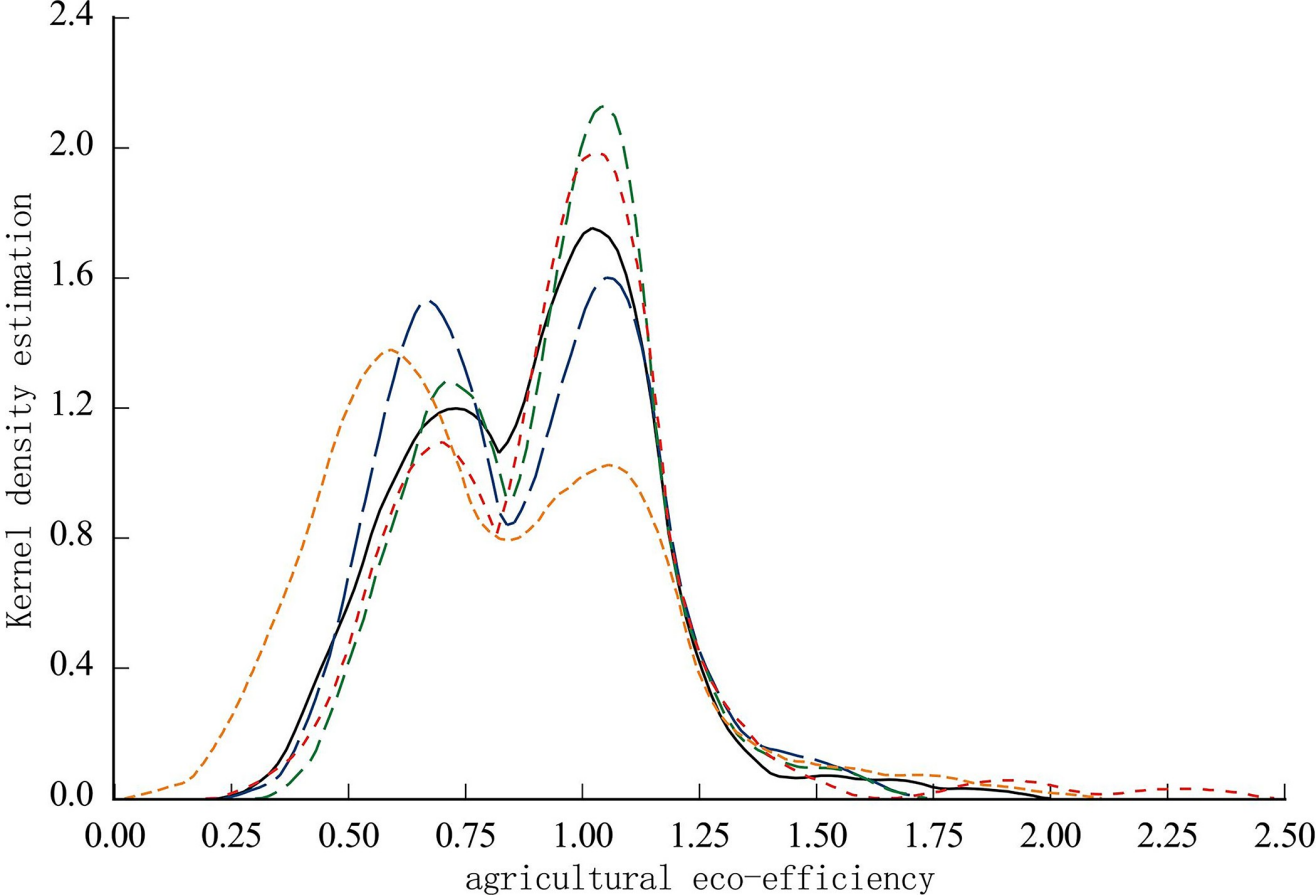
Nuclear density estimation diagram of agricultural ecological efficiency in Hebei Province.

In order to investigate the spatial imbalance state of agricultural ecological efficiency in Hebei Province, this paper used 2005, 2008, 2011, 2014 and 2018 and the Eviews 10 software, drawing nuclear density estimates of agricultural ecological efficiency in Hebei Province. As shown in Fig 5, the horizontal axis represents the value of agricultural ecological efficiency in Hebei Province, and the vertical axis represents the nuclear density value.

According to the time change trend, the nuclear density curves of the five observed time points showed an obvious bimodal distribution, but there were some differences in the peak height, peak width and efficiency value of the central axis of the two peaks. In 2005, the first wave peak was significantly higher than the second wave peak, and the corresponding agricultural ecological efficiency values were 0.59 and 1.06, respectively. The width of the two wave peaks were similar, indicating the agricultural ecological efficiency in Hebei Province in 2005. The number of counties with low efficiency levels was significantly higher than the number of counties with high efficiency levels. In 2008, the first peak and the second peak corresponded to ecological efficiency values of 0.72 and 1.03, respectively. Compared with 2005, the first peaked to the right, the second wave peaked with a small offset to the left, the spacing between the two peaks narrowed, and the second wave peak height was far higher than the first wave height. This showed that, over time, agricultural ecological efficiency showed a convergent development trend in which relatively effective county units gradually increased, and counties gradually shifted to high grade. In 2011, the first peak and the second peak corresponded to ecological efficiency values of 0.73 and 1.04 respectively. Compared with 2008, the peaks were slightly offset to the right, and two peak heights were significantly higher than the previous period. Additionally, the peak width narrowed, showing that the agricultural ecological efficiency value was further improved. In 2014, the M bimodal distribution was presented. The height and width of the first and second wave peaks were similar, and the corresponding agricultural ecological efficiency values were 0.70 and 1.04 respectively, which showed that the agricultural ecological efficiency in Hebei Province showed a relatively balanced polarization phenomenon in which the numbers of counties with high efficiency and low efficiency were equal. In 2018, the ecological efficiency values corresponding to the first and second wave peaks were 0.75 and 1.01, respectively. The height of the second wave was much higher than that of the first wave, and the width of the first wave was greater than the second wave, indicating that over time, the agricultural ecological efficiency in some counties in Hebei Province improved, showing an overall trend of shifting toward high efficiency.

Regarding overall change: a) in terms of shape, the observation points showed bimodal distribution, which indicated that the agricultural ecological efficiency in Hebei Province was highly polarized, and that high efficiency and low efficiency counties were obvious. This indicated an obvious spatial imbalance in agricultural ecological efficiency in Hebei Province. Additionally, b) from the perspective of position, the density curve of the agricultural ecological efficiency was right-shifted, showing the agricultural ecological efficiency of the county. Furthermore, c) from the perspective of peak height, from the initial stage to the end of the study, the first wave peak height decreased first. Then, the second wave peak height first slightly decreased, and then increased, showing an overall significant increasing trend. This showed that agricultural ecological efficiency improved significantly, and the overall development was good. Lastly, d) from the perspective of peak width, compared with the early stage of the study, data indicated that the spatial imbalance of agricultural ecological efficiency decreased during the study period.

In recent years, with gradual improvements in agricultural mechanization levels, increased rural labor released to towns and the enhancement of environmental awareness, the agricultural ecological efficiency in most counties was improved. However, due to natural conditions, agricultural development mechanisms and economic development levels, the agricultural ecological efficiency remained low. Still, there was a clear and ongoing trend of improvement, and the gap with other counties narrowed to a higher level.

(ii) Spatial distribution pattern analysis

**Fig 6:**
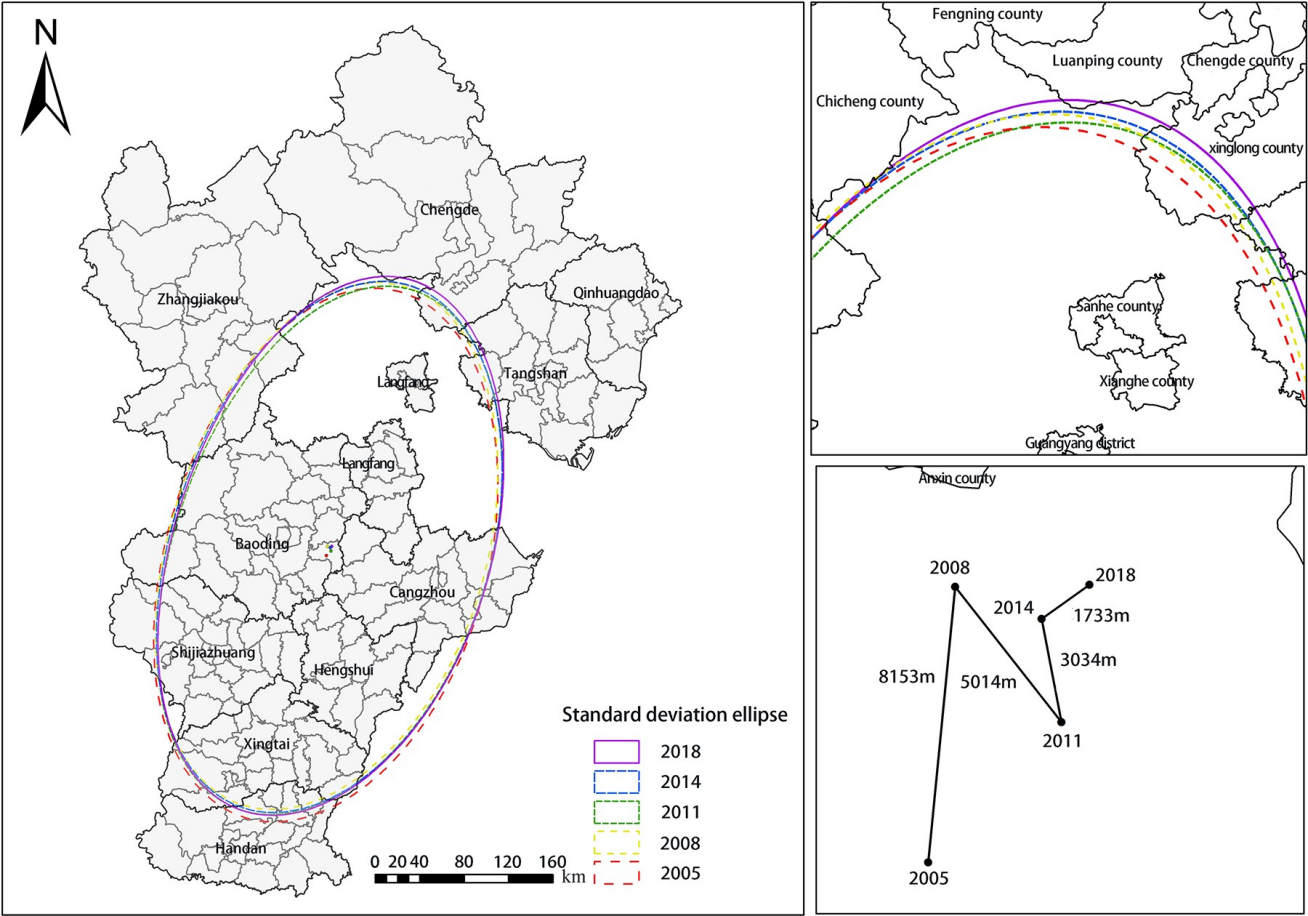
Standard deviation of agricultural ecological efficiency.

Standard deviation ellipses can well reflect the spatial distribution patterns and macroscopic evolutionary laws of agricultural ecological efficiency in Hebei Province. The standard deviation ellipse of ArcGIS 10.3 software was drawn using the direction distribution tool, and relevant parameters, such as the center of gravity coordinate, long axis and short axis, were calculated. The results are shown in Fig 6.

The position of the standard deviation was relatively stable and shifted to the northeast direction. The standard deviation decreased, the length of the short axis decreased, and the shape index decreased toward the northeast. This showed that the spatial distribution of agricultural ecological efficiency in Hebei Province was relatively stable and difficult to change significantly over a short period. Compared with the early research period, the spatial distribution of agricultural ecological efficiency was more concentrated to the south (east), north (west), west (south), and the counties in the east and north of Hebei Province were more obvious. Primary reasons for this could include economic strength, strong support for green agriculture development, the implementation of two-type-oriented agriculture, increasing awareness of environmental protection, and the increasing demand for a better life. These and perhaps other factors combined to improve agricultural ecological efficiency and narrow gaps between counties.

#### 4.2.2 Analysis of the microscopic spatial and temporal evolution characteristics

(i) Traditional Markov transfer matrix

In order to deeply analyze the internal characteristics of the timing evolution of agricultural ecological efficiency in Hebei Province, a traditional Markov transfer probability matrix was constructed. In the evaluation of agricultural eco-efficiency in Hebei Province, agricultural eco-efficiency was divided into four grades, namely low, middle and low, middle and high. Thus, a 4×4 Markov transfer matrix was obtained, represented by n = 1, 2, 3 and 4, respectively. The results are shown in Table 5.

**Table 5:** Markov transfer probability matrix of agricultural ecological efficiency in Hebei Province.

	K	1	2	3	4
**1**	460	0.6565	0.1935	0.137	0.013
**2**	421	0.1544	0.5297	0.2969	0.019
**3**	560	0.0964	0.2161	0.5804	0.1071
**4**	392	0.0077	0.0281	0.1429	0.8214

The probability values on the diagonal in Table 5 represent the possibility of unchanged agroecological efficiency types between adjacent years, reflecting the possibility of shifting between states of agroecological efficiency. According to Table 5, the minimum value on the diagonal was 52.97%, significantly greater than the maximum value on the off-diagonal, 29. 69%, indicating that the transfer of agricultural ecological efficiency types in each state showed strong stability.

There was a club convergence phenomenon in county agricultural ecological efficiency values in Hebei Province. In Table 5, P44 = 0.8214 shows that the possibility of maintaining the high grade in the previous year was 82.14%, while the maximum possibility of downward transfer was P43 = 0.1429; P11 = 0.6565, indicating that the low grade in the previous year was 65.65%. The maximum possibility of upward transfer was P12 = 0.1935. In addition, the elements P11 and P44 at both ends of the main diagonal had the largest values in the whole table, indicating that the agricultural ecological efficiency in Hebei Province remained at the lowest and highest levels; that is, the club convergence phenomenon of high–high agglomeration, low–low agglomeration, high promotion, low inhibition and low suppression in agricultural ecological efficiency grades.

In adjacent years, it was difficult to achieve the cross-level transfer and development of agricultural ecological efficiency in neighboring counties. Often, state transfer occurred on both sides of the diagonal; the so-called cross-level transfer denoted agricultural ecological efficiency transfer (up or down) of two levels or more. As can be seen from Table 5, the transfer probability across levels was low, with a maximum possibility of 13.7% and a minimum of 0.77%. The probability value across levels was much lower than the probability value of the elements on both sides of the diagonal, indicating that changes in agricultural ecological efficiency values are a long-term development process and cannot be completed in a short time.

(ii) The spatial Markov transfer matrix

The traditional Markov transition probability matrix focused only on changes between the states of agricultural ecological efficiency. However, with the increasing degree of openness, the flow of agricultural production factors between counties grew more frequent, and the position of geographical location factors in the regional economic development grew more significant. The spatial lag condition was introduced to construct the transfer probability matrix, and the influence of different geographical location factors on the transfer of agricultural ecological efficiency was deeply explored by comparing the transfer probability of agricultural ecological efficiency under different spatial neighbors. Table 6 shows the spatial Markov transfer probability matrix of agricultural ecological efficiency in Hebei Province.

**Table 6:** Markov transfer probability matrix of agricultural ecological efficiency space in Hebei Province.

		K	1	2	3	4
**1**	1	161	0.6957	0.1925	0.0994	0.0124
	2	97	0.1753	0.5155	0.2784	0.0309
	3	113	0.1416	0.3186	0.4602	0.0796
	4	88	0.0227	0	0.1364	0.8409
**2**	1	123	0.6423	0.2033	0.1545	0
	2	113	0.2035	0.4956	0.2743	0.0265
	3	165	0.0848	0.1636	0.6727	0.0788
	4	55	0.0182	0.1091	0.1455	0.7273
**3**	1	94	0.6064	0.2234	0.1702	0
	2	119	0.084	0.5462	0.3613	0.0084
	3	143	0.0629	0.2098	0.6294	0.0979
	4	103	0	0.0194	0.1748	0.8058
**4**	1	83	0.6627	0.1566	0.1446	0.0361
	2	91	0.1648	0.5604	0.2637	0.011
	3	139	0.1079	0.2014	0.518	0.1727
	4	145	0	0.0138	0.1241	0.8621

Independent analyses of the spatial Markov matrix and the traditional Markov matrix, as well as comparisons of four rules, were obtained. First, the agricultural ecological efficiencies of counties and the neighborhood type were synergistic. Compared with column K in Table 6, when the spatial lag type was 1, the number of counties with low agroecological efficiency level (= 1) at time t was significantly more than that of other classes. Similarly, when the spatial lag type was 4, the number of counties with high agroecological efficiency level (= 4) at time t was also significantly more than that of other classes. Therefore, the number of counties of each type and their geographical neighborhood conditions were synergistic. Second, the geographical neighborhood conditions directly affected the transfer and change of agricultural ecological efficiency in Hebei Province. There were some differences in the transfer probability between counties, with and without considering the geographical neighborhood background. For example, taking the upward transfer of agricultural ecological efficiency type as an example, the P under the geographical neighborhood conditions was not considered 12. At 0.1935, while in the geographical neighborhood of type 3, P12/3 was 0.2234, which increased significantly in terms of state transition probability; P without neighborhood conditions was 34 at 0.1071, while in a geographical neighborhood of type 1, P34/1. At 0.0796, the latter showed a significant decrease in terms of the state transfer probability. Third, agricultural ecological efficiency types in neighboring counties had heterogeneous effects on county state transfer. Adjacent to counties with different agricultural ecological efficiency types, the type transfer probability of a county itself differed. Por instance P23/2 = 0.2743 < P23/3 = 0.3613, meaning the probability of agricultural ecological efficiency moving upward from type 2 to type 3 when adjacent counties were type 2 was much lower than the probability of moving upward from type 2 to type 3 when adjacent counties were type 3; P43/2 = 0.1455 > P43/4 = 0.1241, meaning that the probability of agroecological efficiency moving down from type 4 to type 3 when neighboring counties were type 2 was much greater than the probability of shifting down from type 4 to type 3 when neighboring counties were type 4. Fourth, it effectively explained the club convergence of agricultural ecological efficiency in space. The phenomenon worked as follows: a county with high agricultural ecological efficiency had a significant positive spillover effect, promoting the upward transfer of agricultural ecological efficiency type, inhibiting the downward transfer of agricultural ecological efficiency, and could eventually form a high aggregation situation. On the other hand, for a county with low agricultural ecological efficiency, the negative spillover effect was obvious, and could eventually form a low gathering situation.

### 4.3. Discussion of prediction evolution characteristics

On the basis of agricultural ecological efficiency evaluation and the analysis of macroscopic and microscopic spatial and temporal evolution laws in Hebei Province, we explored whether agricultural ecological efficiency would continue to maintain the existing characteristics in the subsequent long-term evolution process. Evolutionary trend prediction can not only help to grasp the development direction of agricultural ecological efficiency, but also provide a reasonable scientific reference for future governments to introduce corresponding policies to improve agricultural ecological efficiency and promote sustainable development. According to the above studies, the mutual transfer of agricultural ecological efficiency types in Hebei Province was stable enough to maintain the original state. However, this was not absolute stability, but rather the continuous mutual transfer of neighboring grades over time.

The steady-state distribution of the Markov probability transfer is the probability distribution when the transfer between the states reaches a stationary state. It can be used to effectively predict the long-term evolutionary trends of the studied subjects. Generally speaking, the traditional Markov probability transfer matrix was used in n (n) steps, without considering the spatial lag in different geographical neighborhoods, to predict the long-term evolutionary trend of agricultural ecological efficiency in Hebei Province. The prediction results are shown in Table 7.

**Table 7:** Prediction of the long-term evolutionary trend of agricultural ecological efficiency in Hebei Province.

			1	2	3	4
**Spatial lag is not considered**	Initial status		0.251	0.2297	0.3055	0.2139
	Steady-state distribution		0.2109	0.2425	0.3162	0.2304
**Consider space lag**	Steady-state distribution	1	0.2764	0.3747	0.201	0.1478
		2	0.2381	0.2481	0.3799	0.1339
		3	0.1199	0.2565	0.4072	0.2164
		4	0.1747	0.1909	0.2546	0.3798

Excluding the spatial hysteresis, the steady-state distribution was obtained by using Matlab software. In comparison with the initial state, the probability of type 1 decreased from 0.251 to 0.2109; that is, the number of counties in type 1 decreased significantly. The probability of type 2 rose from 0.2297 to 0.2425. The probability of type 3 increased from 0.3055 to 0.3162. The probability of type 4 increased from 0.2139 to 0.2304; that is, the number of counties in types 2, 3, and 4 increased over time, though the increases were different. This showed that, in the long run, agricultural ecological efficiency in Hebei Province gradually shifted upward with time, with more than half of the counties reaching a higher grade than in previous years, and a relatively high grade overall. From the results of the steady state distribution, agricultural ecological efficiency in Hebei Province, after a long-term transfer, did not appear high or low. Types 1, 2 and 3 presented an increasing development trend. The highest probability of type 4 was lower than type 3, demonstrating that, that with the development of agricultural modernization, agricultural ecological efficiency in Hebei Province could gradually reach an effective state, but because of a high drive, low suppression phenomenon and different natural background conditions, it could difficult to achieve large-scale transfer to high-grade counties.

Considering the spatial lag, the steady-state distribution was obtained. When the geographic neighborhood background was at a low level, the number of counties with low and relatively low ecological efficiency values was significantly higher than the traditional steady state distribution. The sum of the number of low and medium-low counties (65. 11%) was nearly twice the sum of the number of middle and high counties (34. 89%), This showed that, in the context of this geographic neighborhood, the ecological efficiency of agriculture in Hebei Province was limited. Space for improvement was also limited; when the geographic neighborhood background was low–medium level, according to the number of counties distributed, Type 3> Type 2> Type 1> Type 4. This showed that county-level agricultural ecological efficiency in Hebei Province has certain potential when the geographic neighborhood background was a medium–high level. The largest number of counties were at that level (type 3), with 40.72% of the total, obviously higher than other types of counties, noted from the gradual disappearance of the bimodal distribution in the traditional state. However, it evolved into a more obvious single peak distribution. When the geographic neighborhood background was at a high level, the steady-state distribution of agricultural ecological efficiency in Hebei Province showed a pattern of increase from low to high counties. This demonstrated the province’s potential for improvement in agricultural ecological efficiency.

## 5. Conclusion

This study used a panel data build evaluation index system, the authors carried out an agricultural ecological efficiency evaluation in Hebei Province. A combination of macroscopic and microscopic methods was used to explore agricultural ecological efficiency in Hebei Province and predict its long-term evolutionary trend, providing a reference for agricultural sustainable development. The study conclusions were as follows:

- On the whole, agricultural ecological efficiency in Hebei Province showed a trend of rising first, then decreasing slightly, and then rising again. In the four regions (eastern Hebei, northern Hebei, central Hebei and southern Hebei), regional agricultural ecological efficiency differed, but the average agricultural ecological efficiency levels more or less increased. Among the regions studied, northern Hebei had the most optimal agricultural ecological efficiency and growth rate. Observing 141 counties in Hebei Province, agricultural ecological efficiency showed a hierarchical cluster development, with the number of low grade, medium–low grade and high grade counties decreasing, while the number of middle and high grade counties increased significantly.
- Macroscopically, the spatial imbalance of agricultural ecological efficiency in Hebei Province was reduced, but the polarization phenomenon always existed. The center of gravity of agricultural ecological efficiency was relatively stable, while the spatial distribution was more concentrated and the direction was more obvious. At the microscopic level, the phenomenon of club convergence was high–high agglomeration, low–low agglomeration, high promotion, low inhibition and low suppression. Moreover, the transfer of agricultural ecological efficiency type showed strong stability. It was difficult to achieve cross-level transfer in adjacent years, and this was significantly affected by geographical neighborhood conditions.
- In terms of evolutionary trends, the long-term evolutionary trend of agricultural ecological efficiency in Hebei Province was relatively optimistic, with great improvement potential. However, it was difficult to achieve large-scale transfer to high-scale counties, whether the influence of geographical space was considered or not. In the long run, without spatial lag, the agricultural ecological efficiency in Hebei Province gradually changed with time evolution, and more than half of the counties reached high and relatively high grades. From the results of steady state distribution, the agricultural ecological efficiency in Hebei Province shifted toward high grade, but it could be difficult to realize the long existence of high drive and low–low inhibition and high suppression and the limitation of different natural background conditions in different counties.

In the construction of the evaluation indicator system, although indicators were selected as comprehensively as possible (referring to previous studies), due to the limitations of data accessibility and existing statistical data, the selected input and output indicators were quantifiable indicators, and the indicators that were difficult to quantify or temporarily lacking were not involved. In the future, econometric models, including contour coefficients, should be used to carry out empirical research on the validity of the Kernel density estimation results. The level of agricultural ecological efficiency is determined, not only by its own input and output, but also by many related factors.
